# The effect of low back pain and neck-shoulder stiffness on health-related quality of life: a cross-sectional population-based study

**DOI:** 10.1186/s12891-020-03871-5

**Published:** 2021-01-05

**Authors:** Gentaro Kumagai, Kanichiro Wada, Hitoshi Kudo, Sunao Tanaka, Toru Asari, Daisuke Chiba, Seiya Ota, On Takeda, Kazushige Koyama, Tetsushi Oyama, Shigeyuki Nakaji, Yasuyuki Ishibashi

**Affiliations:** 1 Department of Orthopaedic Surgery, Hirosaki University Graduate School of Medicine, 5 Zaifu-cho, Hirosaki, Aomori, 036-8562 Japan; 2 Department of Social Medicine, Hirosaki University Graduate School of Medicine, 5 Zaifu-cho, Hirosaki, Aomori, 036-8562 Japan

**Keywords:** Neck-shoulder stiffness, Low back pain, Quality of life, Cross-sectional population-based study

## Abstract

**Background:**

This cross-sectional study sought to determine the neck-shoulder stiffness/low back pain (NSS/LBP) comorbidity rate in a Japanese community population and to compare the quality of life (QOL) in individuals with comorbid NSS/LBP, asymptomatic individuals, and those with symptoms of NSS or LBP alone.

**Methods:**

The sample included 1122 subjects (426 men; 696 women) with NSS and LBP symptoms in the previous 3 months, and were grouped according to NSS, LBP, comorbid NSS and LBP symptoms (Comorbid), or no symptoms (NP). They completed the MOS 36-Item Short-Form Health Survey (SF-36). Health QOL was evaluated by the eight domain scores and the Physical Component Summary (PCS) and Mental Component Summary (MCS) scores after adjusting for age. The primary outcome was to examine the association between NSS/LBP, NSS, or LBP and bodily pain of the eight domains of SF-36. Secondary outcome was to compare health-related QOL among the four groups.

**Results:**

Morbidity was 45.6% for NSS and 51.9% for LBP. Comorbidity affected 23% of men and 33% of women. Comorbid NSS/LBP, NSS, and LBP alone were independently associated with bodily pain after adjusting for potential confounders. Men who exhibited comorbidity had significantly lower MCS scores than asymptomatic men. Women who exhibited comorbidity and LBP had significantly lower MCS scores than those with no symptoms or NSS alone. Women who exhibited comorbidity had significantly lower MCS scores than those with no symptoms or LBP alone.

**Conclusions:**

Comorbidity of the two diseases is prevalent in 23% of the men and 33% of women in the Japanese sample. Although NSS, LBP, and comorbidity were independently associated with QOL in terms of pain, QOL was worse in individuals who exhibited comorbidity than in those without symptoms or with NSS alone.

## Background

Chronic neck pain, neck-shoulder stiffness (NSS), and low back pain (LBP) are serious health problems in the general population [1–3]. The prevalence of neck pain and LBP in the general population is 10–15% [1–4] and 15–45% [5, 6] respectively. Moreover, the three-month prevalence of LBP or neck pain in the US is reported to be 31% (34 million people with only LBP, 9 million with neck pain, and 19 million with both LBP and neck pain) [7]. NSS, which is called “Katakori” in Japanese, is a common ailment in Japan and is characterized by myotonia, heaviness, and dull pain between the cervical and scapular region [8]. Additionally, LBP and NSS are the most frequent complaints among men and women, respectively [9]. Therefore, these symptoms contribute to the economic burden of disease [10, 11], disability [12, 13], absenteeism in the workplace [14], and diminished work capacity [11, 15]. In addition, these health issues often led to long-lasting disability [6].

While several studies have described the link between LBP or neck pain and quality of life (QOL) [16–21], they have not addressed the effect of comorbid neck and LBP symptoms on QOL. Neck pain and LBP symptoms are common to various clinical entities and can occur by themselves or along with other somatic complaints. Several studies suggest that the comorbidity of conditions like shoulder pain and LBP is associated with neck disorders and that adults with previous neck, back, or shoulder injuries are more likely to experience chronic neck pain [3]. Therefore, it is important to clarify the rate of NSS/LBP comorbidity and to determine whether comorbid NSS/LBP adversely affects QOL more than NSS or LBP alone. Accordingly, the present study sought to determine the NSS/LBP comorbidity rate in subjects who participated in a Japanese health promotion program and to compare the QOL of individuals with comorbid NSS/LBP, asymptomatic individuals, and those with symptoms of NSS or LBP alone.

## Methods

### Participants and outline of the research

Subjects for this cross-sectional study were recruited through the Iwaki Health Promotion Project, which provided annual health checkups to the general population in the Iwaki area of Hirosaki city, Japan over a 10-year period. Each year, the program served about 1000 people who lived in the city and were at least 19 years of age [22–24]. Our research on neck pain and LBP complaints was conducted as part of this project.

**Fig. 1:**
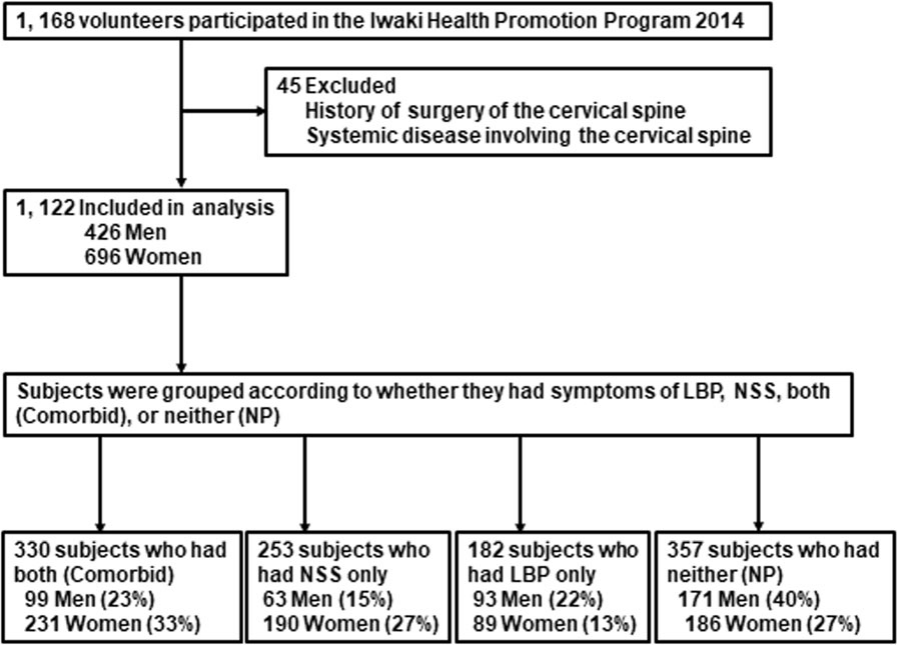
Flow diagram of a cross-sectional study

In 2014, we recruited subjects for this study from the 1167 participants of the community health program. After detailed explanation of the project was provided, participants of the community health program had the option to either take only the regular health examination or to take an extensive physical examination as part of the “Iwaki Health Promotion Project” and enroll in the study. Subjects were excluded from the study if they had a history of spine trauma or a systemic disease affecting the spine, such as rheumatoid arthritis (Fig. 1). We informed the remaining 1122 subjects (426 men and 696 women), verbally and in writing, about the study’s purpose and methods, explained that their anonymity would be protected and that they could withdraw from the study at any time, and obtained their written consent for participation in the study. Informed consent, written or verbal, was obtained from all participants. Subjects completed all the questionnaires described below. This Health Promotion Project was approved by the ethics committee of the Hirosaki University Graduate School of Medicine (2019–1038).

All subjects filled out questionnaires about their medical history and lifestyle, including alcohol use, smoking, fitness habits, occupational history, family history, and QOL; subsequently, they provided information about their clinical history, specifically regarding various diseases, including neck pain or LBP. Anthropometric measurements included height, weight, body mass index (BMI), and body-fat percentage (BFP), which was assessed using a Bioelectrical Impedance Analysis (BIA) scan (MC-190, Tanita, Japan). Each subject was examined by a highly experienced orthopedist to assess the condition and range of motion of the subjects’ knees, hips, elbows, neck, and lower back. All participants completed the MOS 36-Item Short-Form Health Survey (SF-36) [25]. Health-related QOL was evaluated via SF-36 score, which comprises eight domains (vitality, bodily pain, general health, physical function, mental health, and social, physical, and emotional role functioning), as well as the Physical Component Summary (PCS) and Mental Component Summary (MCS) scores, after adjusting for age. The evaluation of the results was done by attributing scores to each question, which were then transformed into a scale ranging from 0 to 100, where a score of 0 indicated low QOL and a score of 100 indicated a high QOL. Each dimension was analyzed separately.

### NSS and LBP

Subjects filled out a questionnaire detailing whether they had experienced neck pain, NSS between the cervical and scapular regions, or LBP in the lumbar regions within the previous three months. Subjects reported the degree of NSS and LBP at the time of assessment using a visual analog scale (VAS) scored 0–100 points (0–100 mm scale), which measured the frequency (daily, 4–5 days/week, 2–3 days/week, or less than 1/week) and prevalence of the aforementioned symptoms [23, 24]. Subjects were grouped according to whether they had symptoms of LBP, NSS, both (comorbid), or neither (NP), and health-related QOL was compared between the four groups and by gender.

### Statistical analysis

Data analysis was performed using SPSS ver. 12.0 J software (SPSS Inc., Chicago, IL, USA). Continuous values were compared between two groups using the Mann–Whitney *U* test. Categorical (or dichotomous) values were analyzed by using a chi-square test. The primary outcome was to examine the association between the incidence of comorbid or separate NSS and LBP and the continuous value of bodily pain score as a pain index of SF-36. To select potential confounders, which were defined as *p*-value < .05, we compared bodily pain between the existence of lifestyle and medical history using the Mann–Whitney *U* test. Single linear regression analyses were conducted using bodily pain as the dependent variable. The independent variables measured the prevalence of NSS or LBP as separate diseases and the prevalence of NSS and LBP comorbidity. To evaluate the adjusted association, forced entry multiple linear regression analyses were conducted using bodily pain as the dependent variable, while using the prevalence of NSS and LBP separately and the prevalence of comorbidity as the independent variables, adjusted by age, gender, and BMI, which were selected from a list of potential confounders. A secondary outcome was to compare health-related QOL among the four groups by analysis of variance (ANOVA) and Tukey’s post-hoc test. For all analyses, a *p-*value < .05 was considered statistically significant. Values of SF-36 represent means ± standard error of the mean (s.e.m.)

## Results

As shown in Table 1, the age of women was significantly higher (55.3 ± 15.3 years, mean ± SD) than that of men (52.6 ± 15.5 years; *P* = 0.003), as was previously described in detail [24], while the mean BMI was significantly higher in men (*P* <  0.001). Significantly more men than women reported that they drank alcohol (*P* <  0.001), had diabetes (*P* = 0.047), or took diabetes medication (*P* = 0.011), whereas significantly more women reported working (*P* <  0.001), hyperlipidemia (*P* = 0.004), and use of sleeping pills (0.015) and lipid-lowering medication (*P* = 0.001).

**Table 1:** Characteristics of study participants^a^

	All (*n* = 1122)	Men (*n* = 426)	Women (*n* = 696)	*P* value^b^
Age, y ^c^	54.2 ± 15.4	52.6 ± 15.5	55.3 ± 15.3	0.003*
BMI, kg/m^2^	22.7 ± 3.4	23.6 ± 3.1	22.2 ± 3.4	< 0.001*
Highest level of education, *n* (%)				
Primary school	6 (0.5)	3 (0.7)	3 (0.1)	
Middle school	208 (18.5)	69 (16.2)	139 (20.0)	
High school	602 (53.7)	249 (58.5)	353 (50.7)	
Junior college or vocational school	214 (19.1)	58 (13.6)	156 (22.4)	
Graduate degree	83 (7.4)	45 (10.5)	38 (5.5)	
Other	9 (8.0)	2 (0.5)	7 (1.1)	
Lifestyle and medical history, *n* (%)^d^				
Working	1082 (96.4)	395 (92.7)	687 (98.7)	< 0.001^#^
Smokes	129 (11.5)	40 (9.3)	89 (12.8)	0.474
Uses alcohol	481 (42.9)	290 (68.1)	191 (27.4)	< 0.001^#^
Diabetes	58 (5.2)	30 (7)	28 (4)	0.047^#^
Hypertension	282 (25.1)	98 (23)	184 (26.4)	0.436
Hyperlipidemia	145 (12.9)	37 (8.7)	108 (15.5)	0.004^#^
Depression	5 (0.4)	0 (0)	5 (0.7)	0.163
Antidiabetic medication	56 (5.0)	30 (7)	26 (3.7)	0.011^#^
Antihypertensive medication	277 (24.7)	96 (22.5)	181 (26)	0.108
Lipid-lowering medication	141 (12.6)	36 (8.5)	105 (15.1)	0.001^#^
Analgesic medication	44 (3.9)	12 (2.8)	32 (4.6)	0.091
Sleeping pills medications	37 (3.3)	7 (1.6)	30 (4.3)	0.015^#^

^a^Previously described in detail [1]. ^b^Significant differences between men and women are indicated by *P* < 0.05, by *Mann–Whitney *U* or ^#^chi-square test. ^c^Age and BMI are shown as mean ± S.D. ^d^The number (%) of subjects with each characteristic

The prevalence of NSS was significantly higher in women (60.3%) than in men (38.0%, *P* <  0.01; Table 2). NSS prompted more women (9.2%) than men (4.7%) to seek medical assessment, but this difference was not significant (Table 2). The prevalence of LBP was 45% in men and 46% in women; however, this difference was not significant (Table 3). LBP prompted more men (15.3%) than women (14.7%) to seek medical assessment, but this difference was not significant (Table 3). The prevalence of comorbid NSS/LBP was 23% in men and 33% in women (Fig. 1).

**Table 2:** Neck-shoulder stiffness (NSS)

	Men (*n* = 426)	Women (*n* = 696)	*P* value
NSS prevalence, *n* (%)^a^	162 (38.0)	423 (60.3)	< 0.001^#^
VAS of NSS^b^	36.2 ± 23	37.4 ± 22	0.558
NSS symptom frequency, *n* (%)			
Daily	45 (10.6)	126 (18.1)	
4–5 days/week	15 (3.5)	59 (8.5)	
2–3 days/week	15 (3.5)	59 (8.5)	
Less than 1/week	46 (10.8)	105 (15.1)	
Symptoms medically assessed, *n* (%)	20 (4.7)	64 (9.2)	0.379

^a^Subjects who experienced NSS at least once in the 3 months prior to assessment. ^b^Visual analog scale (VAS) results, shown as mean ± SD

^#^chi-square test

**Table 3:** Low back pain (LBP)

	Men (*n* = 426)	Women (*n* = 696)	*P* value
LBP prevalence, *n* (%)^a^	192 (45.1)	320 (46.0)	0.805
VAS of LBP^b^	31.0 ± 21.1	31.6 ± 19.8	0.530
LBP symptom frequency, *n* (%)			
Daily	58 (13.6)	93 (13.4)	
4–5 days/week	18 (4.2)	37 (5.3)	
2–3 days/week	46 (10.8)	92 (13.2)	
Less than 1/week	70 (16.4)	98 (14.1)	
Symptoms medically assessed, *n* (%)	65 (15.3)	102 (14.7)	0.697

^a^Subjects who experienced LBP at least once in the 3 months prior to assessment. ^b^Visual analog scale (VAS) results, shown as mean ± SD

The value of bodily pain was significantly lower in subjects who had hypertension, took antihypertensive medication, analgesic medication, and sleeping pills (Table 4). We selected these medical histories as potential confounders. Single linear regression analyses showed that the prevalence of comorbidity and of LBP by itself were positively associated with bodily pain (Table 5). The prevalence of NSS separately was negatively associated with bodily pain (Table 5). Multiple linear regression analyses showed that comorbid NSS/LBP, NSS, and LBP were independently associated with bodily pain after adjusting for potential confounders (NSS alone, β = − 0.073, 95% CI [− 2.906, − 0.293]; LBP alone, β = 0.143, 95% CI [2.126, 5.047]; comorbid, β = 0.243, 95% CI [3.791, 6.087]; Table 5). The standardized regression coefficient for the association with bodily pain was stronger for comorbidity than for NSS and LBP separately.

**Table 4:** Comparison of bodily pain between the prevalence of lifestyle or medical history

	Existence	Bodily pain^a^	*P* value
Lifestyle and medical history			
Working	Yes (*n* = 1077)	48.7 ± 9.1	0.848
	No (*n* = 45)	50.7 ± 9.3	
Smokes	Yes (*n* = 129)	50.2 ± 9.1	0.962
	No (*n* = 993)	50.8 ± 8.6	
Uses alcohol	Yes (*n* = 481)	50.6 ± 9.4	0.492
	No (*n* = 641)	50.0 ± 9.2	
Diabetes	Yes (*n* = 58)	48.7 ± 9.1	0.336
	No (*n* = 1064)	50.7 ± 9.3	
Hypertension	Yes (*n* = 281)	48.7 ± 9.1	0.006*
	No (*n* = 841)	50.7 ± 9.3	
Hyperlipidemia	Yes (*n* = 145)	49.5 ± 9.1	0.276
	No (*n* = 977)	50.4 ± 9.2	
Depression	Yes (n = 5)	50.2 ± 9.3	0.509
	No (*n* = 1117)	52.5 ± 9.3	
Antidiabetic medication	Yes (*n* = 56)	48.6 ± 10.0	0.349
	No (*n* = 1066)	50.3 ± 9.2	
Antihypertensive medication	Yes (*n* = 277)	48.7 ± 9.1	0.002*
	No (*n* = 845)	50.7 ± 9.3	
Lipid-lowering medication	Yes (*n* = 141)	49.7 ± 9.3	0.546
	No (*n* = 981)	50.3 ± 9.0	
Analgesic medication	Yes (*n* = 44)	42.2 ± 6.3	< 0.001*
	No (*n* = 1078)	50.5 ± 9.2	
Sleeping pills medications	Yes (*n* = 37)	45.9 ± 9.2	0.004*
	No (*n* = 1085)	50.3 ± 9.2	

^a^Bodily pain results, shown as mean ± SD. *Mann–Whitney *U*

**Table 5:** Single and multiple regression analysis relative to bodily pain ^*a*^

Logistic regression (Bodily pain)		*B*	β	95% CI	*P* value
NSS alone	Crude	−2.282	−0.103	−3.573 to −0.992	0.001
	Adjusted	−1.600	−0.073	− 2.906 to − 0.293	0.016
LBP alone	Crude	4.275	0.170	2.823 to 5.728	< 0.001
	Adjusted	3.587	0.143	2.126 to 5.047	< 0.001
Comorbid	Crude	5.055	0.249	3.901 to 6.209	< 0.001
	Adjusted	−4.939	0.243	3.791 to 6.087	< 0.001

B, unstandardized regression coefficient; β, standardized regression coefficient: *r*^2^, coefficient of determination (adjusted)

^a^Single and forced entry multiple regression analysis was performed using bodily pain as the dependent variable and age, Gender, BMI, selected potential confounders (hypertension, antihypertensive medication, and sleeping pills medications), the prevalence of NSS, LBP alone, and comorbid NSS/LBP as independent variables

**Fig. 2:**
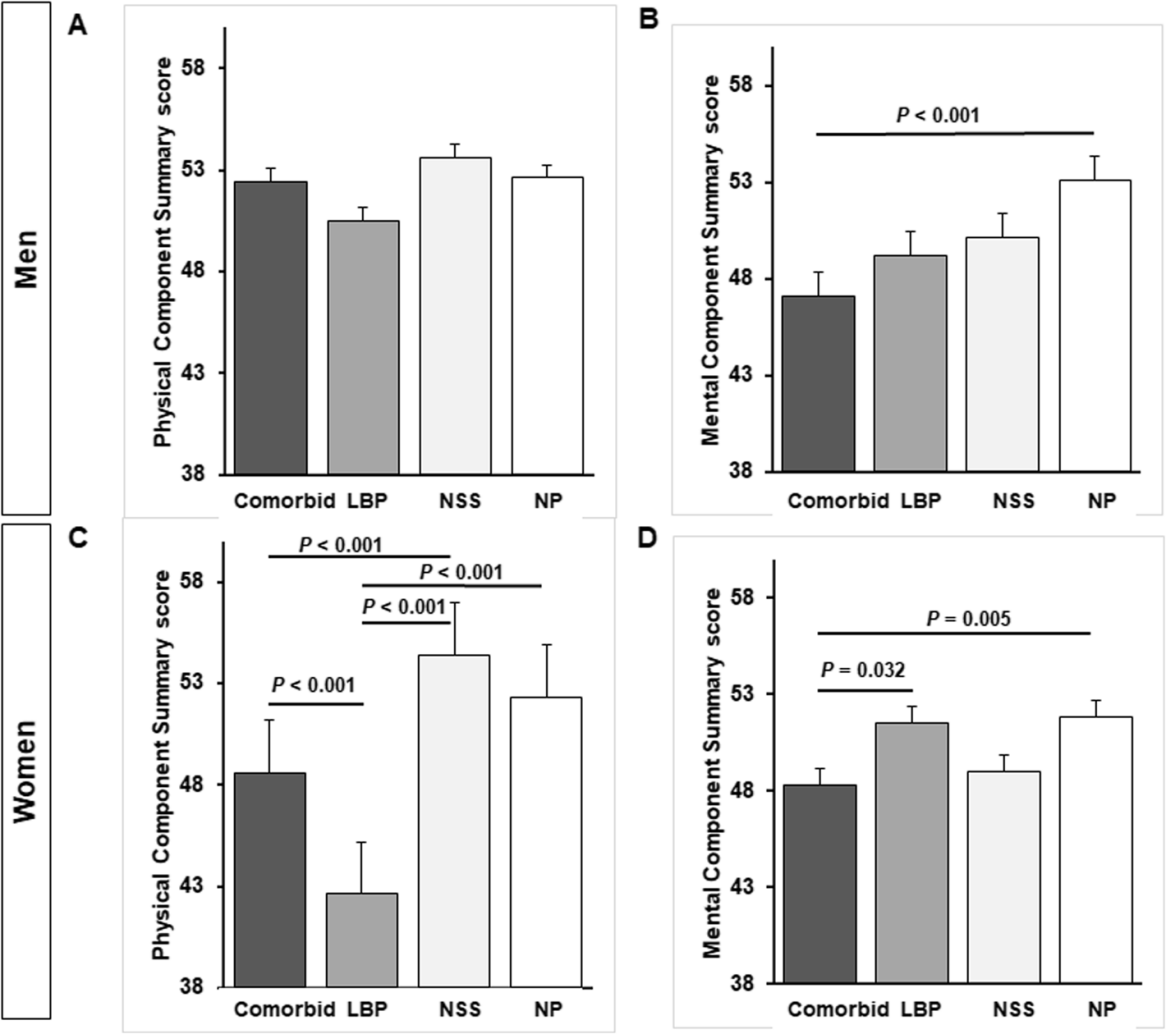
Health-related QOL summaries by gender and symptom group (**a**, **b**). SF-36 PCS and MCS in men who had NP or had NSS, LBP, or comorbid NSS/LBP (**c**, **d**). Differences in PCS and MCS by symptom group in women. Values represent means ± s.e.m.

**Fig. 3:**
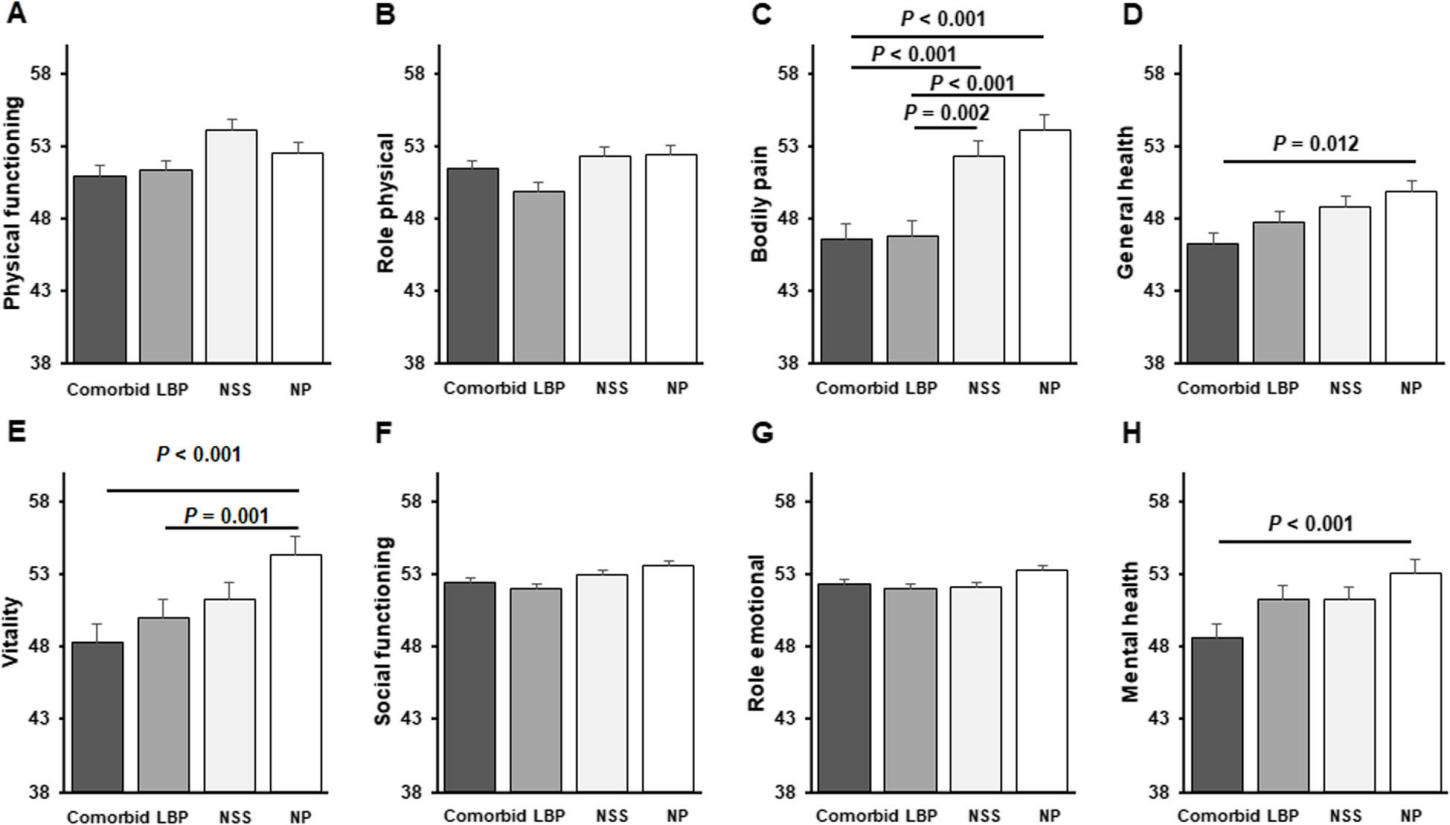
QOL scores in men according to symptom group (**a**–**h**). SF-30 scores for physical functioning, physical role functioning, bodily pain, general health, vitality, social functioning, emotional role functioning, and mental health in men who had NP or had NSS, LBP, or comorbid NSS/LBP. Values represent means ± s.e.m.

**Fig. 4:**
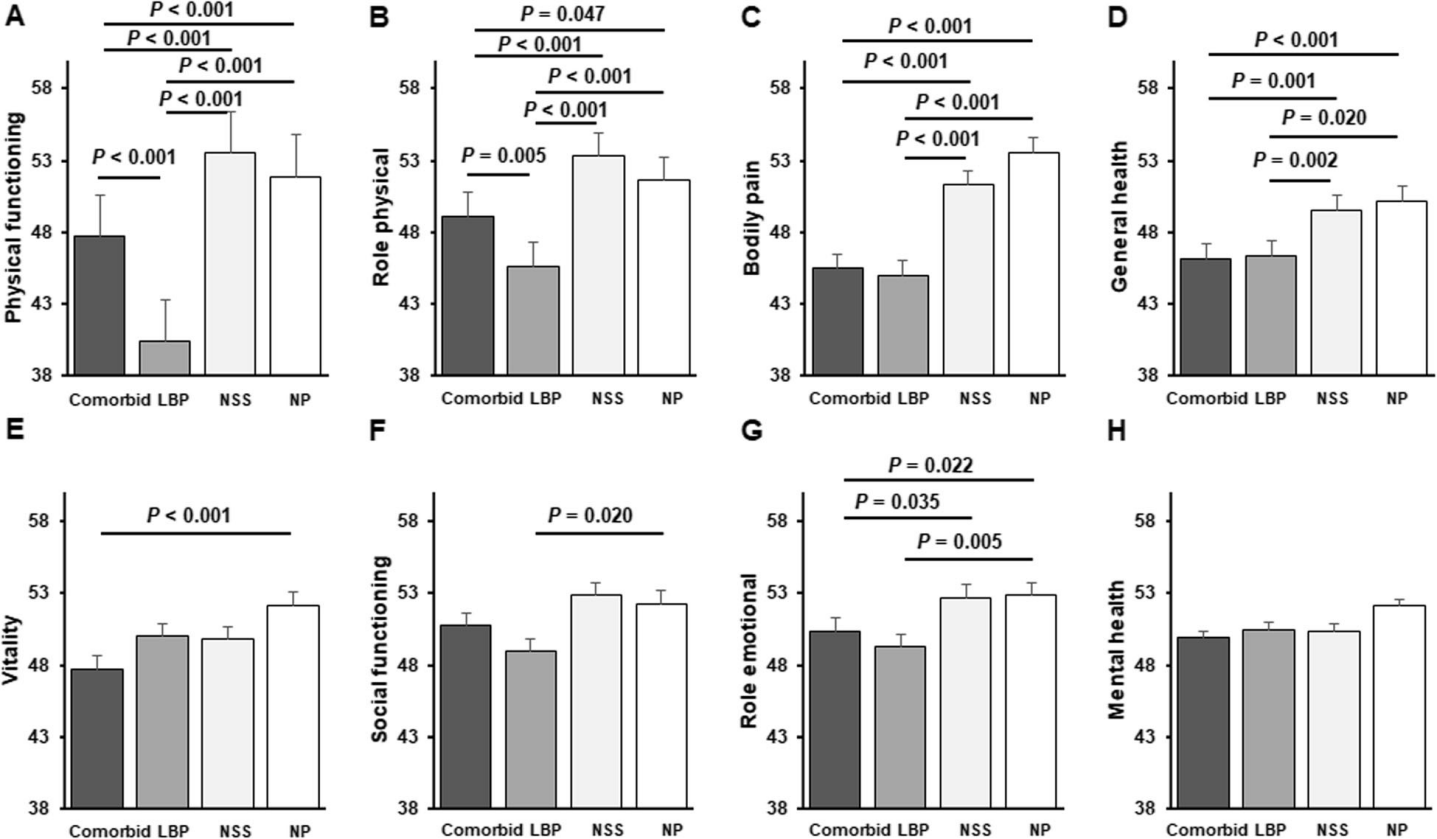
QOL scores in women according to symptom group (**a**–**h**). SF-30 scores for physical functioning, physical role functioning, bodily pain, general health, vitality, social functioning, emotional role functioning, and mental health in women who had NP or had NSS, LBP, or comorbid NSS/LBP. Values represent means ± s.e.m.

Among men, MCS score was significantly lower among those who had comorbid NSS/LBP, than among those in the NP group (*P* <  0.001; Fig. 2). Among women, PCS score was significantly lower in the LBP group than in any of the other groups (*P* <  0.001, Fig. 2) and was significantly lower in the comorbid group than in the NSS group (*P* <  0.001, Fig. 3). Moreover, MCS score was significantly lower in the comorbid group than in the LBP (*P* = 0.032) and NP groups (*P* = 0.0005, Fig. 1). Men in the comorbid group had significantly lower scores for bodily pain (*P* <  0.001), general health (*P* = 0.012), vitality (*P* < 0.001), and mental health (*P* < 0.001) than those in the NP group (Fig. 3). Among women, the scales of physical functioning, physical role functioning, bodily pain, general health, vitality, and emotional role functioning were significantly lower in the comorbid group than in the NSS or NP groups (Fig. 4). Similarly, these scores were significantly lower in the LBP group than in the NSS or NP groups, especially the physical functioning score (Fig. 4).

## Discussion

We found that 23% of men and 33% of women in a Japanese sample had comorbid NSS and LBP. The prevalence of NSS and LBP separately as well as the prevalence of these diseases together were independently associated with bodily pain after adjusting for potential confounders. QOL was worse in subjects with comorbid NSS/LBP and among those with LBP separately, compared with asymptomatic subjects or those with symptoms of NSS alone.

Kumagai et al. reported a significantly higher prevalence of NSS among Japanese women (37.3%, *n* = 477), compared with men (2.8%; *n* = 284) [23]. Similarly, another Japanese study reported that NSS which had a prevalence of 48.3%, was more common in women than in men and was most common among individuals aged 20–50, decreasing with age thereafter [20]. Moreover, Muraki et al. reported an incidence of low back pain (LBP) of 28.3% in men and 31.2% in women (8.6 and 9.5% per year, respectively) in a large-scale population of a nationwide cohort study in Japan [26]. Thus, the prevalence of NSS and LBP in our study was similar to that in previous reports. We found that the rate of NSS/LBP comorbidity was 23% in men and 33% in women. A cross-sectional study of 21,225 twins who completed a web-based questionnaire for comorbid LBP and neck-shoulder pain (NSP) showed that 13% of women and 7% of men had a higher prevalence [27, 28]. Genetic factors had a considerably greater influence on the occurrence of comorbid LBP and NSP [28]. Similar to previous studies, comorbid rates for women were higher than those of men. High rates of NSS and LBP comorbidity in a general population highlight the importance of considering the effect of comorbidities when studying these symptoms separately.

QOL was worse in subjects with comorbid NSS/LBP and those with LBP alone than in asymptomatic subjects or those with symptoms of NSS alone. Moreover, PCS scores, in particular, physical functioning and role physical was worse in subjects with LBP alone than other subjects in women. Many studies suggest that neck pain and LBP reduce physical activity [29] and working capacity [11], cause disability [12, 13], and result in occupational absenteeism [30]. Accordingly, neck pain and LBP are negatively related to QOL [16]. However, neck pain, although less disabling than LBP [31], may still have a considerable impact on QOL. Studies in Japanese communities report that NSS is associated with pain in the upper extremities and lower QOL scores but not with pain in the lower extremities or medical complications [20], and that LBP and knee pain significantly affect individuals’ QOL [21]. As expected, comorbid NSS/LBP and LBP alone were more strongly associated with QOL than NSS alone in linear regression analyses after adjusting potential confounders. The relationship between LBP or comorbid NSS/LBP and QOL, in particular bodily pain, should be considered in the context of lifestyle. In our study population, the high rate of employment (92.7% of men and 98.7% of women) may have affected LBP but not NSS, with a consequent effect on physical-health QOL in women. A longitudinal study with the same individuals may provide clues to the underlying cause of their clinical symptoms.

This study has several limitations that should be noted. First, we did not evaluate the chronicity, location, and distribution of neck pain and LBP symptoms. Particularly, chronicity should be evaluated in a future longitudinal study. Second, our study population was geographically limited to a farming village district with a high percentage of older adults. Therefore, lifestyle aspects, such as occupations and hobbies, should be considered in future studies. Third, pain in the cervical and lumbar spine can be due to a variety of causes, such as facet degeneration, lateral recess stenosis, foraminal stenosis, and herniation. Fourth, we did not examine radiographic data. Finally, the number of male subjects having neck or lumbar symptoms was relatively small. Regarding bodily pain, power analysis demonstrated that 586 male subjects would be needed to assess the statistically significant difference between NSS and NS group (G*Power, ver. 3.1.9.3; α error: 0.05, 1-β error: 0.80, and one tail). Future large-scale cohort studies could clarify the detailed pathology of neck and lumbar symptoms.

## Conclusions

In summary, subjects with comorbid NSS and LBP (23% of men and 33% of women) had a lower QOL than asymptomatic subjects or subjects with NSS alone. Moreover, NSS/LBP comorbidity was associated with worse mental health in both men and women. NSS/LBP comorbidity also decreased physical-health QOL in women. Our current findings can be considered in differential diagnosis and in devising strategies to prevent nonspecific neck pain or LBP symptoms**.**

## Data Availability

The datasets used and/or analysed during the current study are available from the corresponding author on reasonable request.

## Abbreviations

NSS: Neck-shoulder stiffness
LBP: Low back pain
QOL: Quality of life weight
BMI: Body mass index
BFP: Body-fat percentage
BIA: Bioelectrical Impedance Analysis
SF-36: 36-Item Short-Form Health Survey
PCS: Physical Component Summary
MCS: Mental Component Summary
VAS: Visual analog scale
ANOVA: Among the four groups by analysis of variance
